# The dynamic relationships between the active and catabolic vitamin D metabolites, their ratios, and associations with PTH

**DOI:** 10.1038/s41598-019-43462-6

**Published:** 2019-05-06

**Authors:** Jonathan C. Y. Tang, Sarah Jackson, Neil P. Walsh, Julie Greeves, William D. Fraser, Bioanalytical Facility team, Nicole Ball, John Dutton, Holly Nicholls, Isabelle Piec, Christopher J. Washbourne

**Affiliations:** 1 Norwich Medical School, University of East Anglia, Norwich Research Park, Norwich, UK; 2 HQ Army, Andover, UK; 3 College of Human Sciences, Bangor University, Bangor, UK; 4 Departments of Diabetes, Endocrinology and Clinical Biochemistry, Norfolk and Norwich University Hospital NHS Foundation Trust, Colney Lane, Norwich UK; 5 Bioanalytical Facility, Bob Champion Research and Education Building, University of East Anglia, Norwich Research Park, Norwich, UK

**Keywords:** Calcium and vitamin D, Epidemiology

## Abstract

Vitamin D status, assessed by serum concentration of 25(OH)D, is the prime candidate marker for many disease-association studies, but the interplay between the subsequent 1,25-dihydroxyvitamin D (1,25(OH)_2_D) and 24,25-dihydroxyvitamin D (24,25(OH)_2_D) metabolites is unclear. In this study, we conducted an analysis from a large cohort of healthy, physically fit, young army recruits (n = 940). We found a significant, inverse relationship between serum 25(OH)D and 1,25(OH)_2_D:24,25(OH)_2_D vitamin D metabolite ratio (VMR) (r^2^Exp = 0.582, p < 0.0001), and demonstrated a significant association with increasing PTH concentration (p < 0.001). Circannual rhythms were evident for all vitamin D metabolites and VMRs except for 1,25(OH)_2_D when fitted to Cosinor curves. We estimated 1,25(OH)_2_D:24,25(OH)_2_D VMR of ≥35 to be the threshold value for vitamin D insufficiency, and ≥51 to be predictive of vitamin D deficiency. Our three-dimensional model provides mechanistic insight into the vitamin D-PTH endocrine system, and further substantiates the role of 24,25(OH)_2_D in human physiology. The model sets a new paradigm for vitamin D treatment strategy, and may help the establishment of vitamin D-adjusted PTH reference intervals. The study was approved by the UK Ministry of Defence research ethics committee (MODREC 165/Gen/10 and 692/MoDREC/15). ClinicalTrials.gov Identifier NCT02416895.

## Introduction

The vitamin D pathway is a dynamic system, and its functional role in bone health and other diseases is the subject of intense research. Associations of vitamin D deficiency with a wide spectrum of disease states have drawn attention from the scientific community and increasing awareness of the general population. Despite vitamin D deficiency being a global public health concern, the approach through improving vitamin D status by supplementation, dietary intake and increased sunlight exposure, has resulted in mixed outcomes^1–3^. The contradictory evidence has prompted studies on the metabolites of vitamin D. The most abundant metabolite in circulation is 25-hydroxyvitamin D (25(OH)D), which exists in two major forms: 25-hydroxycholecalciferol (25(OH)D3) and 25-hydroxyergocalciferol (25(OH)D2). Measurement of serum total 25(OH)D (D3 + D2) is the barometer of vitamin D status; concentrations ≤30 nmol/L and between 30–50 nmol/L are defined as deficient and insufficient, respectively by the U.S Institute of Medicine (IOM)^4^ and the UK National Osteoporosis Society (NOS)^5^. 1,25-dihydroxyvitamin D (1,25(OH)_2_D) is synthesised by the hydroxylation of 25(OH)D through the actions of 1α-hydroxylase produced in the renal tubules. 1,25(OH)_2_D is the most biologically active form of vitamin D and circulates in pmol/L concentration; it controls intestinal absorption of calcium and phosphate, stimulates osteoclast activity, and helps regulate the release of parathyroid hormone (PTH). Although 1,25(OH)_2_D is derived from 25(OH)D, there is no direct correlation in serum concentrations between the two vitamin D metabolites except in patients with chronic kidney disease (CKD)^6^, where a greater association is observed between 1,25(OH)_2_D and 25(OH)D, dependent upon the severity of the renal impairment. The lack of a direct relationship, despite their close proximity in the metabolic pathway, is due to the tight regulation of the hydroxylation enzymes expressed by the actions of CYP27B1 and CYP24A1. CYP24A1 produces 24-hydroxylase that converts 25(OH)D into 24,25-dihydroxyvitamin D (24,25(OH)_2_D). The transcription of the *CYP24A1* gene is stimulated by the phosphate-regulating hormone fibroblast growth factor-23 (FGF23), and when PTH is suppressed. The combination results in an increase in serum 24,25(OH)_2_D. We have previously described a concentration-dependent relationship between serum 25(OH)D and 24,25(OH)_2_D^7^. We have also reported a patient, presenting with idiopathic infantile hypercalcaemia (IIH), who was diagnosed with biallelic *CYP24A1* mutations resulting in the inability to produce 24,25(OH)_2_D from 25(OH)D, and had an elevated serum 1,25(OH)_2_D and a persistent state of hypercalcaemia. The use of 25(OH)D:24,25(OH)_2_D vitamin D metabolite ratio (VMR) can be a valuable tool in identifying such pathological conditions resulting from impaired CYP24A1 function^8,9^. The use of VMR in the population can provide an assessment of the vitamin D catabolic status; thus allowing a targeted approach to vitamin D supplementation^10^.

In this report, we describe a novel approach to the interpretation of serum 25(OH)D and 1,25(OH)_2_D concentrations that incorporates 24,25(OH)_2_D values. Using data from a large cohort of young healthy adults as our reference population, we report the intricate relationships between active and catabolic forms of vitamin D metabolites, and the influence on PTH.

## Results

Results from 940 participants were included in the data analysis. Statistical analyses on 25(OH)D and 24,25(OH)_2_D were performed on the respective total (sum of D3 + D2) values, the distributions were untrimmed and no outlier was removed. Summary of the distribution of biochemical profile is shown in Table 1. 25(OH)D2 was found in 57.8% of the subjects, mean (range) of 4.2 nmol/L (0.6–29.1). 24,25(OH)_2_D2 was found in 0.4% of the subjects, mean (range) 1.5 nmol/L (1.2–1.8).

**Table 1:** Distribution of biochemical measurements performed in the study.

Profile	mean	SD	Min	2.5^th^ Percentile	25^th^ Percentile	Median	75^th^ Percentile	97.5^th^ Percentile	Max
25(OH)D, nmol/L	62.4	29.8	6.9	18.1	39.8	59.2	81.0	130.9	222.5
24,25(OH)_2_D, nmol/L	5.4	3.3	0.5	1.0	2.9	4.9	7.4	13.0	29.6
1,25(OH)_2_D, pmol/L	138.8	39.6	32.3	71.9	111.0	135	161.0	229.7	380.0
25(OH)D:24,25(OH)_2_D VMR	13	4	2	7	10	12	15	25	39
1,25(OH)_2_D:24,25(OH)_2_D VMR	38	33	5	9	18	28	45	132	300
Intact PTH, pmol/L	3.7	1.2	1.0	1.9	2.9	3.5	4.3	6.8	11.4
ACa, mmol/L	2.38	0.07	2.00	2.20	2.32	2.40	2.41	2.50	2.60

### 24,25(OH)_2_D and 25(OH)D

**Figure 1:**
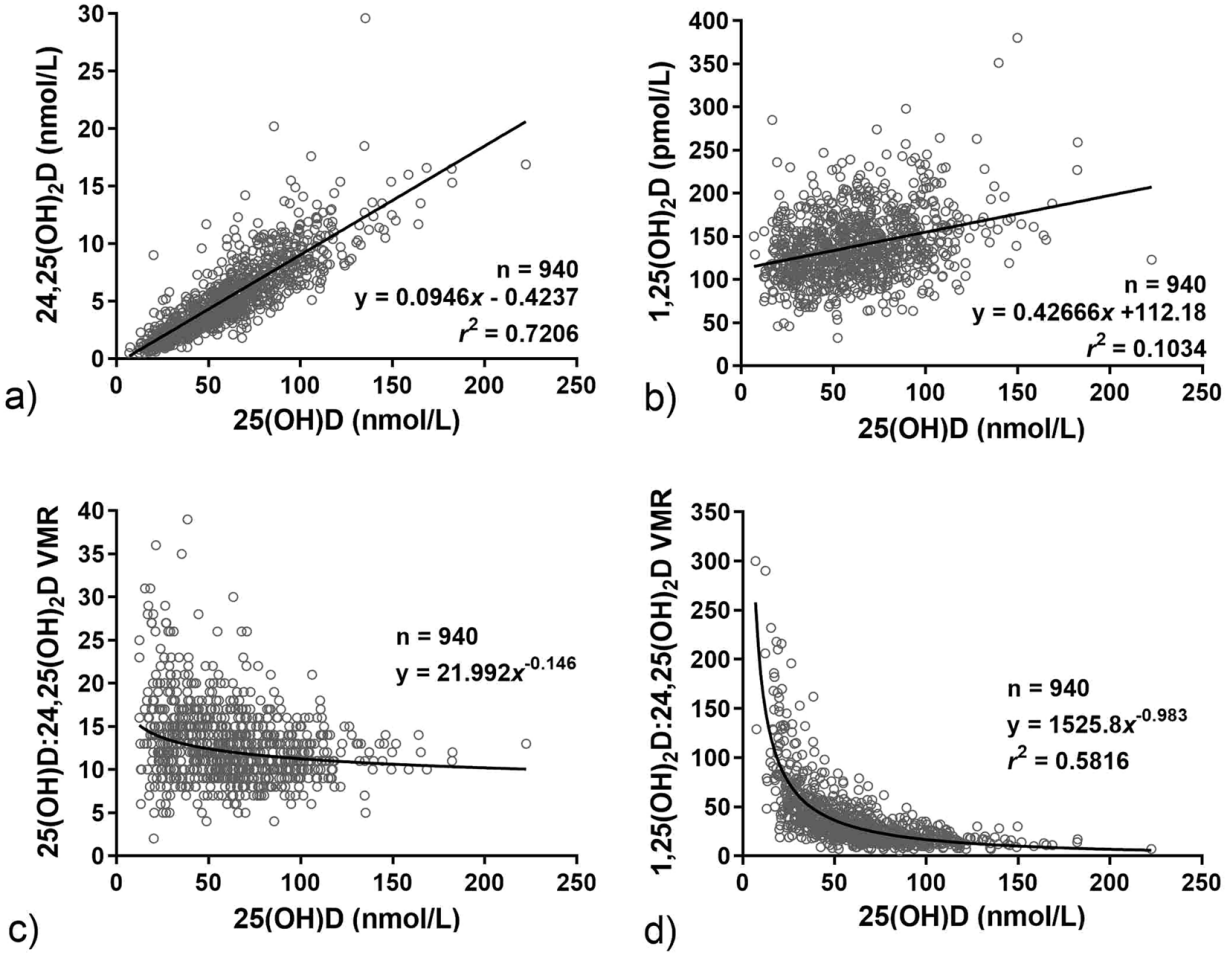
Non-parametric correlations of (**a**) 24,25(OH)_2_D (**b**) 1,25(OH)_2_D (**c**) 25(OH)D:24,25(OH)_2_D VMR and (**d**) 1,25(OH)_2_D:24,25(OH)_2_D VMR, against their respective 25(OH)D concentration. Solid lines in (**a**,**b**) represent linear regression line. LOWESS fitted curve in (**c**,**d**) (99% point fit). The mean 24,25(OH)_2_D and 1,25(OH)_2_D concentrations, 25(OH)D:24,25(OH)_2_D VMR and 1,25(OH)_2_D:24,25(OH)_2_D VMR represent 8.7%, 222.4%, 20.8% and 60.9% of their respective 25(OH)D concentration. Assay lower limit of quantification (LLoQ): 25(OH)D and 24,25(OH)_2_D = 0.1 nmol/L, 1,25(OH)_2_D = 12 pmol/L.

The mean concentration of 24,25(OH)_2_D was on average 9.5-fold lower than 25(OH)D. Linear regression analysis (Fig. 1a) showed a directly proportional relationship between 24,25(OH)_2_D and 25(OH)D concentrations: $[24,25{({\rm{O}}{\rm{H}})}_{2}{\rm{D}}]\,=\,0.0946\,\times \,[25({\rm{O}}{\rm{H}}){\rm{D}}]\,-\,0.42;\,{r}^{2}\,=\,0.7206$. Using this equation, we derived serum 24,25(OH)_2_D concentration of ≥4.3 nmol/L to be equivalent to the IOM vitamin D replete status (i.e. 25(OH)D of 50 nmol/L), and 24,25(OH)_2_D concentration of ≤2.4 nmol/L is equivalent to deficiency status (i.e. 25(OH)D of ≤30 nmol/L).

### 1,25(OH)_2_D and 25(OH)D

Despite a direct enzymatic conversion of 25(OH)D to 1,25(OH)_2_D, we showed no correlation in serum concentrations between these two vitamin D metabolites (Fig. 1b). This finding is consistent with published studies; 1,25(OH)_2_D is able to directly inhibit the expression of 1α-hydroxylase, and indirectly inhibit by suppressing PTH and stimulating FGF23 production^11,12^. This negative feedback system provides an essential safeguard mechanism against hypercalcaemia, hence 1,25(OH)_2_D concentration is unaffected by the circulatory concentration of 25(OH)D.

### 25(OH)D:24,25(OH)_2_D VMR and vitamin D status

**Figure 2:**
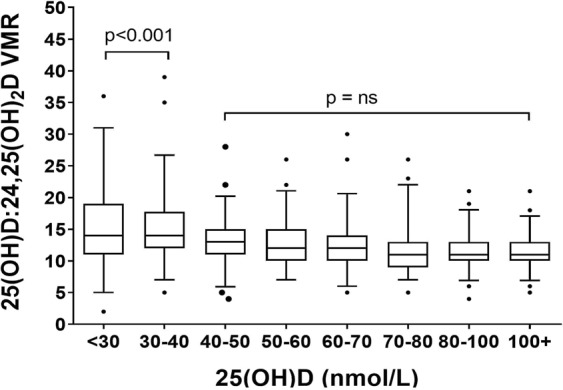
Distribution of 25(OH)D:24,25(OH)_2_D VMR by 25(OH)D intervals. Each interval contains an equal number of subjects to illustrate the significantly elevated ratio in those with serum 25(OH)D ≤ 50 nmol/L. Box and whiskers represent the median, interquartile range and 95% population intervals.

The 25(OH)D:24,25(OH)_2_D VMR showed an indirect relationship with 25(OH)D (Fig. 1c); LOWESS fitting showed a steady increase in 25(OH)D:24,25(OH)_2_D VMR with the decline in 25(OH)D concentration. One-way ANOVA showed a significant increase in 25(OH)D:24,25(OH)_2_D VMR (p > 0.001) at 25(OH)D below 50 nmol/L (Fig. 2). The greatest increase was observed when 25(OH)D concentration decreased below ≤30 nmol/L. The decrease in relative production of serum 24,25(OH)_2_D in response to the decline in 25(OH)D suggests down-regulation of CYP24A1.

### 1,25(OH)_2_D:24,25(OH)_2_D VMR and vitamin D status

**Figure 3:**
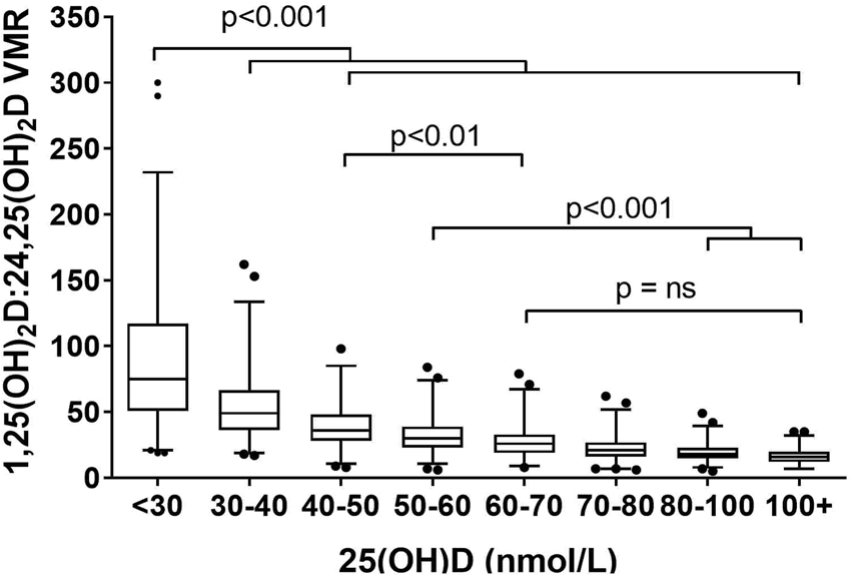
Distribution of 1,25(OH)_2_D:24,25(OH)_2_D VMR by 25(OH)D intervals. It demonstrates the exponential increase in 1,25(OH)_2_D:24,25(OH)_2_D VMR with the decrease in serum 25(OH)D. Box and whiskers represent the median, interquartile range and 95% population intervals. Each interval contains an equal number of subjects.

**Figure 4:**
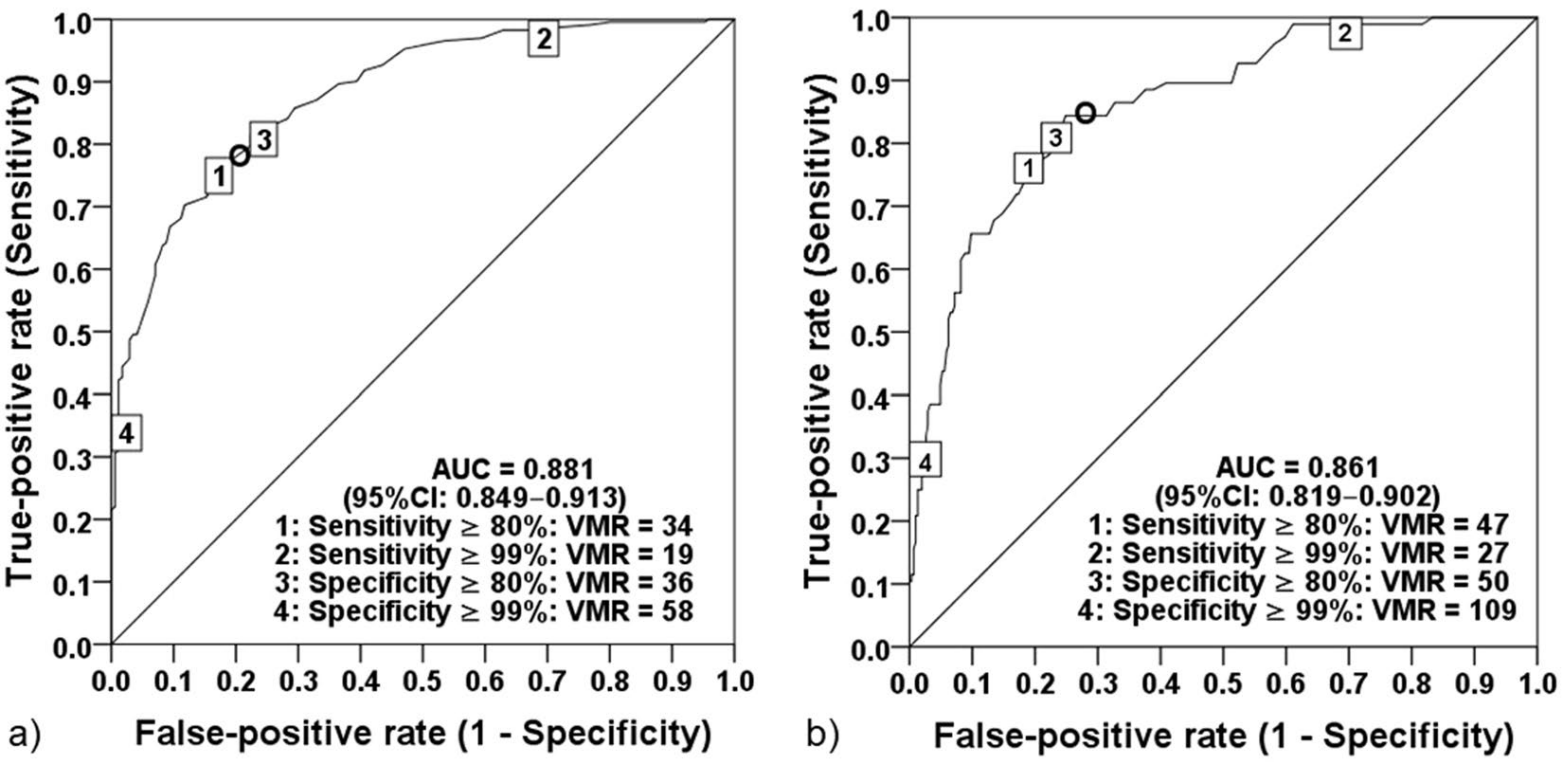
Diagnostic performance of 1,25(OH)_2_D:24,25(OH)_2_D VMR in the assessment of vitamin D status during winter months (Jan–April) (n = 402). Receiver Operating Characteristic (ROC) curve depicts diagnostic sensitivity and specificity levels. (**O**) represents decision threshold for (**a)** vitamin D replete (i.e. 25(OH)D ≥ 50 nmol/L), 1,25(OH)_2_D:24,25(OH)_2_D VMR threshold value of 35 (sensitivity = 80%, specificity = 78%), (**b)** vitamin D insufficiency (i.e. 25(OH)D ≥ 30 nmol/L), 1,25(OH)_2_D:24,25(OH)_2_D VMR threshold value of 51 (sensitivity = 82%, specificity = 74%). The diagonal lines represent the line of no discrimination.

Vitamin D status, as indicated by 25(OH)D concentrations, revealed an exponential negative correlation (r^2^Exp = 0.582) with 1,25(OH)_2_D:24,25(OH)_2_D VMR (Fig. 1d). *Post hoc* analysis identified a significant increase in 1,25(OH)_2_D:24,25(OH)_2_D VMR at 25(OH)D ≤ 60 nmol/L (Fig. 3). Using the Jacobson and Truax^13,14^ method to determine the cut-off value for clinically significant change^7^, we estimated 1,25(OH)_2_D:24,25(OH)_2_D VMR of ≥35 to be the predictive threshold value for vitamin D insufficiency, and ≥51 to be predictive threshold for vitamin D deficiency. The threshold values were determined from subject samples collected in the winter months (January to April) due to the seasonal variation of 25(OH)D. Receiver Operating Characteristic (ROC) curves generated from data collected between January to April produced area under the curve (AUC) values of 0.88 and 0.86, indicating the VMR cut-offs are excellent at discriminating individuals with vitamin D insufficiency and deficiency. The 1,25(OH)_2_D:24,25(OH)_2_D VMR at 35 and 51 achieved true positive rate (sensitivity) at 80% and 78%, respectively, and false positive rate (specificity) of 82% and 74%, respectively (Fig. 4a,b).

### 1,25(OH)_2_D:24,25(OH)_2_D VMR and PTH

Circulating PTH is influenced by 25(OH)D and 1,25(OH)_2_D, and *vice versa*. We hypothesised that PTH concentration changes with 1,25(OH)_2_D:24,25(OH)_2_D VMR and 25(OH)D. To test our hypothesis, median PTH concentrations were established from grid analysis based on groupings of 1,25(OH)_2_D:24,25(OH)_2_D VMR and 25(OH)D in ascending order (Table 2). A decrease in PTH concentration was observed from the high 1,25(OH)_2_D:24,25(OH)_2_D VMR (100+) and low 25(OH)D (<30 nmol/L) group, to the low 1,25(OH)_2_D:24,25(OH)_2_D VMR (<30) and high 25(OH)D (100+ nmol/L) group. Using Kruskal-Wallis independent non-parametric analysis to test the distribution of PTH across all groups, a highly significant (p > 0.001) change in PTH concentration was found across the 1,25(OH)_2_D:24,25(OH)_2_D VMR and 25(OH)D categories, hence the null hypothesis was rejected. Using Spearman’s rank correlation coefficient (2-tailed) to assess monotonic functions between variables, significant positive correlations were evident between VMRs and PTH (1,25(OH)_2_D:24,25(OH)_2_D VMR rho = 0.249, p > 0.001 and 25(OH)D:24,25(OH)_2_D VMR rho = 0.134, p > 0.001); whereas vitamin D metabolites showed significant negative correlations with PTH (25(OH)D rho = −0.287, p > 0.001, 24,25(OH)_2_D rho = −0.282, p > 0.001 and 1,25(OH)_2_D rho = −0.87, p > 0.001). The statistical significance remain unchanged after adjustment for BMD and BMI as covariates.

**Table 2:** Median (SEM) PTH concentrations in categories of increasing 1,25(OH)_2_D:24,25(OH)_2_D VMR and 25(OH)D. One-way ANOVA showed PTH concentrations decreased significantly (p > 0.001) from high 1,25(OH)_2_D:24,25(OH)_2_D VMR/low 25(OH)D to low 1,25(OH)_2_D:24,25(OH)_2_D VMR/high 25(OH)D.

Median PTH, pmol/L		25(OH)D, nmol/L			
		<30	30–50	51–100	100+
1,25(OH)_2_D:24,25(OH)_2_D VMR	100+	5.7 (0.2)*	5.4 (0.5)*	—	—
	51–100	4.3 (0.2)*	3.8 (0.2)	3.4 (0.3)	—
	30–50	4.0 (0.2)*	3.7 (0.1)	3.5 (0.1)	2.7 (0.2)*
	<30	3.8 (0.3)	3.9 (0.1)	3.3 (0.1)*	3.2 (0.1)*

*Denotes significance at the p < 0.05 level.

### Circannual variations in vitamin D metabolites and VMRs

**Figure 5:**
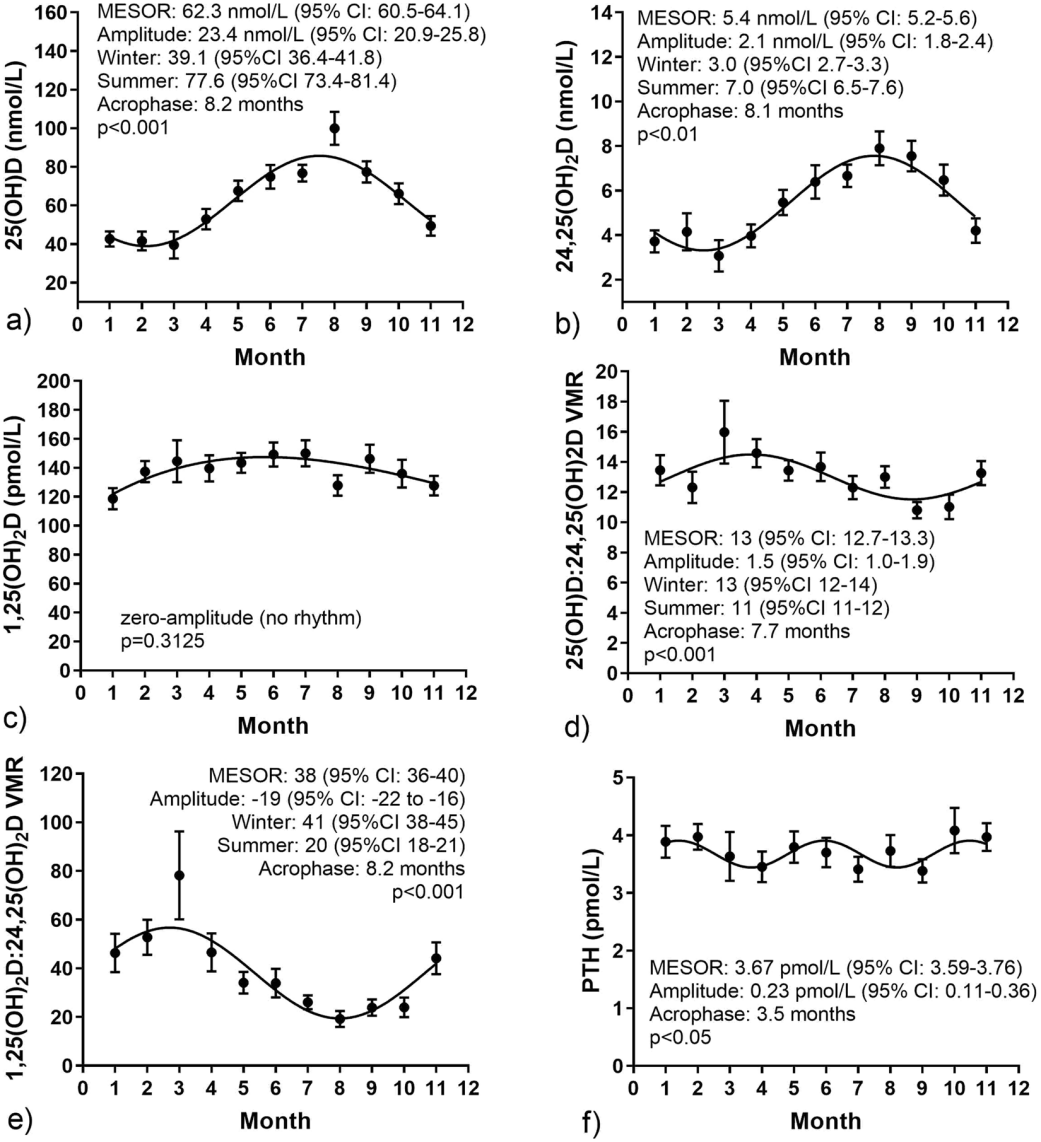
Cosinor-fit circannual rhythm for (**a)** 25(OH)D, (**b)** 24,25(OH)_2_D, (**c)** 1,25(OH)_2_D, (**d)** 25(OH)D:24,25(OH)_2_D, (**e)** 1,25(OH)_2_D:24,25(OH)_2_D, (**f)** PTH. Error bars represent 95% CI.

Cosinor-fit curves (Fig. 5) show significant circannual rhythm for 25(OH)D (p < 0.001), 24,25(OH)_2_D (p < 0.01), 25(OH)D:24,25(OH)_2_D VMR (p < 0.001), 1,25(OH)_2_D:24,25(OH)_2_D VMR (p < 0.001) and PTH (p < 0.05). No significant rhythm was observed for 1,25(OH)_2_D (p = 3.125). The rhythm observed for 25(OH)D is consistent with previous reports^15,16^. 24,25(OH)_2_D showed a similar peak (July–Aug) and nadir (Jan–Mar) pattern as for 25(OH)D. 25(OH)D:24,25(OH)_2_D VMR and 1,25(OH)_2_D:24,25(OH)_2_D VMR exhibited patterns in the opposite direction, with peak (Mar–April) and nadir (Aug–Sept) suggesting that the production of 24,25(OH)_2_D is relatively higher during summer/early autumn months. Acrophase, defined as the lag time between rhythm-adjusted mean and peak cycle value, was on average (SD) of 8.1(0.3) months for all vitamin D metabolites except for 1,25(OH)_2_D. A low amplitude, circasemiannual PTH secretory rhythm was observed, with an acrophase of 3.5 months.

## Discussion

This is the first study to demonstrate a relationship between serum concentrations of 25(OH)D and 1,25(OH)_2_D when expressed as a relative ratio with serum 24,25(OH)_2_D. We provide evidence that the conversion of 25(OH)D to 1,25(OH)_2_D is associated with the catabolism of 25(OH)D to 24,25(OH)_2_D, which can be assessed by the measurement of serum 24,25(OH)_2_D and its derived VMR.

The inverse exponential correlation between 1,25(OH)_2_D:24,25(OH)_2_D VMR and 25(OH)D provides insight into the dynamics of vitamin D metabolites in healthy, young adults; when vitamin D status is sufficient, serum concentrations of 1,25(OH)_2_D and 24,25(OH)_2_D are maintained in relative proportion and showed no significant change beyond the sufficient threshold. In contrast, when vitamin D status is insufficient, a progressive and highly significant increase in 1,25(OH)_2_D:24,25(OH)_2_D VMR is evidence that the production of serum 1,25(OH)_2_D is favoured over 24,25(OH)_2_D as the availability of vitamin D precursors in circulation diminishes. Our data imply two possible regulatory functions of the 24,25(OH)_2_D pathway; in hypervitaminosis, the pathway is ‘switched on’ to allow excess 25(OH)D to be converted to 24,25(OH)_2_D. The 24-hydroxylase pathway results in the formation of calcitroic acid for excretion. In hypovitaminosis, the 24,25(OH)_2_D pathway is partially inactivated to conserve 25(OH)D and to maintain an adequate supply of substrate for conversion to 1,25(OH)_2_D. Although the biological activity of 24,25(OH)_2_D is yet to be fully elucidated, its role in vitamin D catabolism appears certain. Low serum concentrations of 24,25(OH)_2_D and elevated 25(OH)D:24,25(OH)_2_D VMR is useful in identifying patients with loss-of-function *CYP24A1* mutations^8,9,17^. In our previous publication^7^, we described a case of biallelic *CYP24A1* mutation in a patient presenting with hypercalcaemia, elevated serum 1,25(OH)_2_D concentration (293 pmol/L, reference range 43–144 pmol/L), and elevated 25(OH)D:24,25(OH)_2_D VMR of 32. On diagnosis, the patient’s 1,25(OH)_2_D:24,25(OH)_2_D VMR was 212 (1.6 times the upper 97.5th percentile of 132), which was attributed to supplementation with vitamin D. One month after treatment for hypercalcaemia and cessation of vitamin D supplement, serum 1,25(OH)_2_D was within the reference range, the 1,25(OH)_2_D:24,25(OH)_2_D VMR decreased to 130 (below the 97^th^ percentile), and the 25(OH)D:24,25(OH)_2_D VMR remained elevated at 35.

A major finding of this study was the link between the vitamin D metabolites and VMRs with the distribution of PTH. To the best of our knowledge, this is the first such report in a human population study. It is widely accepted that the PTH concentration is associated with 25(OH)D, but not with the active 1,25(OH)_2_D. This is due to the tight regulatory mechanisms, and the regulatory processes that take place via the vitamin D receptor (VDR) to activate intracellular transport of calcium and stimulate PTH secretion. Using our 1,25(OH)_2_D:24,25(OH)_2_D VMR and 25(OH)D model (Fig. 1d), we have shown that individuals with low 25(OH)D (≤50 nmol/L), normal 1,25(OH)_2_D but high 1,25(OH)_2_D:24,25(OH)_2_D VMR (≥101) have significantly higher PTH concentration than those at the opposite end of the spectrum. An interpretation of our finding supports a biological role of 24,25(OH)_2_D other than as a catabolic metabolite of vitamin D. Relative high production of 24,25(OH)_2_D may reduce the bioactivity of 25(OH)D and 1,25(OH)_2_D, particularly extra-renal production of 1,25(OH)_2_D, to down-regulate the secretion of PTH whilst maintaining 1,25(OH)_2_D concentrations within the strict boundaries required for appropriate calcium homeostasis. Relatively low 24,25(OH)_2_D could enhance the anabolic effects of vitamin D metabolism, by stimulating PTH production. The biological action of 24,25(OH)_2_D on the inhibition of PTH secretion was first reported in animal and *in vitro* models in the late seventies^18,19^. More recently there is increasing evidence supporting physiological functions of 24,25(OH)_2_D on bone and cartilage^20–22^ in promoting fracture healing, and protection against cartilage damage. The existence of a 24,25(OH)_2_D-specific nuclear or membrane receptor has been reported^23^, but its function has yet to be elucidated. Given that CYP24A1, the enzyme responsible for the production of 24,25(OH)_2_D, is present in most tissues with VDR, understanding the mechanisms controlling the production of 24,25(OH)_2_D relative to other vitamin D metabolites may have significance beyond vitamin D catabolism, potentially shaping vitamin D supplementation strategies.

Mapping the circannual rhythms of vitamin D metabolites and VMRs is an important component of this study. We have published reports on longitudinal studies (Macdonald *et al*.)^15,16,24^, decribing the changes in serum 25(OH)D and 24,25(OH)_2_D throughout a year in vitamin D supplemented or non-supplemented subjects. In the VICtORy (Vitamin D and CardiOvascularRisk)^15^ and VICtORy RECALL^16^ randomised controlled studies performed using a group of postmenopausal women residing in the northeast of UK, the placebo group showed a two-fold increase in serum 25(OH)D in peak summer months (July–August), compared to the nadir in late winter months (January–March). Our younger cohort of healthy individuals in this study showed similar trends; 24,25(OH)_2_D had a propensity to fluctuate with 25(OH)D throughout the year, with changes between summer and winter months, as indicated by a lower 25(OH)D:24,25(OH)_2_D VMR during January to March than during July to September. Serum 1,25(OH)_2_D displayed no rhythm and was within the reference range throughout the year. The circannual variation of 1,25(OH)_2_D:24,25(OH)_2_D VMR was dependent on 24,25(OH)_2_D, with a peak-to-nadir difference of 19; such sharp demarcation between seasons would inevitably create uncertainty when using 1,25(OH)_2_D:24,25(OH)_2_D VMR in diagnostic decision-making. In contrast, 25(OH)D:24,25(OH)_2_D VMR is less susceptible to seasonal fluctuation, allowing the use of the VMR with fixed reference intervals irrespective of the time of the year.

One strength of our data is the chosen cohort; with participants attending blood sampling visits at strictly controlled time intervals and that the vitamin D metabolites were measured using gold-standard methodologies. The participants are well-defined, largely from a similar social-economic background, and exposed to the same level of fitness training, diet, and frequency of outdoor activities. The relative homogeneity of the subjects of our study population in combination with our inclusion criteria allowed us to confidently form a reference population and identify important changes in analytes. The limitations are that our findings are observational and based on baseline sampling at the start of training. Also, our cohort represents young adults of Caucasian extraction (92.9%), and cannot be extrapolated to the wider population of mixed ethnicity. The predictive threshold values were established based on the equivalent vitamin D status as described by IOM, and not based on our own data as the study was not randomly controlled. We did not measure vitamin D binding protein (VDBP) and free 25(OH)D due to the ethnic homogeneity of our population, and we did not exclude factors that may influence VDBP levels (e.g. oral contraceptive use in female recruits). We also acknowledge the use of VMR in reverse (i.e. 24,25(OH)_2_D:25(OH)D - as described in^25^) can be an alternative approach to express the relationship. Lastly, the sample data used in the current study is a subset of the Army cohort used to establish the reference intervals of serum 24,25(OH)_2_D^7^. Whilst there is a degree of overlap, the overall content of this report, analytical approaches and conclusions described are independent of the previous report.

In conclusion, the present analysis characterises the absolute and relative concentrations of the active and catabolic form of vitamin D metabolites in a well-defined young, healthy and physically fit population. The use of VMRs provides insight into the metabolic pathway and the variations exhibited throughout the year. We propose a three-dimensional model incorporating 1,25(OH)_2_D, 24,25(OH)_2_D and 25(OH)D measurements and report a strong correlation between metabolites that are linked with PTH. Such modelling could help establish vitamin D-adjusted PTH reference intervals, and ultimately contribute to the goal of a “Treat to target” approach to vitamin D supplementation.

## Methods

### Study design

The study received ethics approval from the UK Ministry of Defence Research Ethics Committee and was conducted in accordance with the Declaration of Helsinki (2013). The characteristics of the subjects included in the study are shown in Table 3. In total, 2252 new British Army recruits at the start of phase one training volunteered for the study. Written informed consent was obtained from all study participants, and each required to complete a detailed health questionnaire, including medical history and the use of supplements. All recruits undertook physical and cognitive testing, and a detailed medical examination prior to joining the army. The British Army entry requirements restrict individuals with chronic medical conditions; therefore, our study population represents a medically screened, disease-free, and physically fit population. In the analysis, we excluded individuals who reported the use of calcium and vitamin D supplements (including multivitamins and cod liver oil), and excluded participants who reported injury and illness prior to recruitment; conditions such as being underweight, eating disorders, or those with a history of bone fracture. 940 participants were included in the final statistical analyses. The majority of participants were from Caucasian population (92.9%), with a minority from a diverse ethnicity (Asian 1.6%, Black 1.7%, Chinese 0.1%, mixed 3%, others 0.7%).

**Table 3:** Baseline characteristics of the subjects included in the study.

n	Male	Female
	652	288
Mean age, years (range)	21.7 (18–32)	22.1 (18–32)
Height, m	1.77 (6.4)	1.66 (5.9)
body mass, kg	75.9 (9.8)	64.7 (7.5)
Body mass index (BMI)	24.1 (2.6)	23.4 (3.3)
Total body BMD (g/cm^2^)	1.24 (0.10)	1.16 (0.09)

*Data shown in mean ± SD otherwise stated.

### Sample collection

Blood samples were collected during the period of June 2014 and March 2016. Venous blood samples were obtained from the participants at the start of 14-week long basic military training. Sample collections were scheduled on a monthly basis to balance the seasonal variations. Each intake comprised, on average (range), 86 (43–120) participants. Blood samples were collected into serum gel separator tube and EDTA plasma container (BD Vacutainer). Samples were centrifuged immediately after collection at 3,000 × g for 10 minutes. Plasma/serum layers were aliquoted into a separate polystyrene tube and stored at −20 °C until analysis. All samples were anonymised to the researchers at the point of access.

### Ethical approval

The study was approved by the UK Ministry of Defence research ethics committee (MODREC 165/Gen/10 and 692/MoDREC/15). ClinicalTrials.gov Identifier NCT02416895.

### LC-MS/MS measurements of serum 25(OH)D and 24,25(OH)_2_D

Liquid chromatography tandem mass spectrometry (LC-MS/MS) was performed as described^7,26–28^. The method quantified 25(OH)D3, 25(OH)D2, 24,25(OH)_2_D3 and 24,25(OH)_2_D2 simultaneously from a single injection. 25(OH)D3 and 25(OH)D2 were calibrated using commercial standards (Chromsystems, München, Germany) traceable to standard reference material SRM972a from the National Institute of Science and Technology (NIST)^25,29^, and showed linearity between 0–200 nmol/L. The inter/intra-assay coefficient of variation (CV) was ≤9%, the lower limit of quantification (LLoQ) of 0.1 nmol/L. The assay showed <8% accuracy bias against NIST reference method on the Vitamin D external quality assessment (DEQAS) scheme. 24,25(OH)_2_D3 and 24,25(OH)_2_D2 were calibrated using in-house spiked standards traceable to NIST SRM972a. The assay is linear between 0–25 nmol/L; inter/intra-assay CV was ≤11%, LLoQ of 0.1 nmol/L for 24,25(OH)_2_D3 and 0.8 nmol/L for 24,25(OH)_2_D2.

### Measurements of serum 1,25(OH)_2_D

The DiaSorin LIAISON^®^ XL 1,25(OH)_2_D chemiluminescent immunoassay (Stillwater, MN, USA) method was used. The sandwich assay utilises a recombinant fusion protein for the capture of 1,25(OH)_2_D molecule and a murine monoclonal antibody detection system. The assay measures total 1,25(OH)_2_D between 12–480 pmol/L, the inter/intra-assay CV was ≤9.2%. The mean assay recovery was 94 ± 2%. On the Vitamin D external quality assessment (DEQAS) scheme, the assay showed ≤8.5% bias against method-specific mean and ≤9.1% bias against all method mean.

### Biochemical analysis

Intact PTH and albumin-adjusted calcium (ACa) were analysed on the COBAS^®^ (Roche Diagnostics, Mannheim, Germany) platform. PTH in EDTA plasma was measured using electrochemiluminescence immunoassay (ECLIA), the inter-assay CV was ≤3.8% across the analytical range of 1.2–5000 pg/mL. Total calcium and albumin were measured based on spectrophotometric methods. The inter-assay CV for Ca was ≤1.6%, albumin was ≤1.1%. ACa value is calculated using the equation $ACa=(\,-\,0.8\times [Albumin]-4)+[Total\,Ca]$.

### Statistical analysis

Descriptive statistics, scatterplots, ROC and LOWESS curves were constructed and analysed by Statistical Package for the Social Science (SPSS) version 22.0.0.1 (IBM, New York, USA) and GraphPad Prism 7 (GraphPad, San Diego, CA, USA). Univariate and multivariable linear regression analyses and one-way ANOVA were used to estimate associations. LOWESS curve fitting was used to explore nonlinear relationships between variables. Kruskal-Wallis independent analysis and Spearman’s rho were used to establish associations in non-parametric variables. Statistical significance was defined as p < 0.05. Frequency distribution histograms of the data were visually examined and checked for transcriptional and pre/post analytical errors before exclusion for statistical analysis. Confidence interval (CI) was established at 95% of the population. Circannual rhythm analysis was performed by population-mean cosinor analysis, based on cosinor-fitting equation $y={\rm{MESOR}}+{\rm{Amplitude}}\times \,\cos ({\rm{Frequency}}(x)+{\rm{acrophase}})$. Midline estimate statistic of rhythm (MESOR), defined as the rhythm-adjusted mean value. Acrophase is the difference (time) between MESOR and peak value in the cosine curve.

## Data Availability

The datasets generated and analysed during the current study are not publicly available due to data protection. Data request is subject to approval by the UK Ministry of Defence.
